# Knowledge, attitude and practice towards risky sexual behaviors among secondary and preparatory students of Metu town, south western Ethiopia

**DOI:** 10.1186/s12889-020-09371-4

**Published:** 2020-09-14

**Authors:** Terefe keto, Ayele Tilahun, Aklilu Mamo

**Affiliations:** 1 Department of Comprehensive Clinical Nursing, Mizan Aman Health Science College, Mizan, Ethiopia; 2 Department of Nursing, College of Health Sciences, Mizan Tepi University, Mizan, Ethiopia

**Keywords:** Adolescents, Attitude, Knowledge, Practice, Risky sexual behaviors, Metu

## Abstract

**Background:**

Sexual risk behaviors are defined as sexual activities that may make an individual liable to the risk of sexually transmitted infections including Human Immunodeficiency virus (HIV) and unplanned pregnancies. Adolescents are at high risk of developing sexual risk behavior. The rate of risky sexual behaviors and the spread of STIs continue to be increase among the adolescent population. Therefore this study aimed to assess Metu secondary and preparatory school adolescent knowledge, attitude, and practice of risky sexual behaviors.

**Methods:**

Institution based descriptive cross sectional study was conducted among Metu secondary and preparatory school students from 04 Feb 2019–07 June 2019. The study participants were selected through systematic random sampling techniques and the data was collected through self-administered questionnaires. A Total of 361 study subjects were included in the study. Data was entered in EpiData and analyzed by using SPSS version 21.

**Results:**

Three hundred sixty one respondents participated in this study. Of those, 75.9% of them have awareness about risky sexual behaviors and about 76.5. % of them has awareness on consequences of unsafe sex. Among the total study participants, about 22.7% of them had previously practiced in sexual activity; of these 61.7% of respondents had more than one sexual partner. From the respondents who had practiced sex, 19.8% of them had always used condom during their sexual intercourse with their partners, while 58% of them never used condom during sexual intercourse.

**Conclusions:**

Even though the majority of the students have an awareness regarding sexual risk behaviors, a considerable number of students have practiced risky sexual behaviors that might predispose them for different sexual and reproductive health problems and peer pressure was revealed as a major factor that influences the respondents towards their first sexual intercourse. Peers have greater influence on the positive and negative behavior of their friends. Therefore the school should emphasize on promoting peer educators and peer discussion to protect adolescents and youth from risky sexual behaviors.

## Background

Different organizations and scholars define adolescents in different ways. The World Health Organization (WHO) defines adolescence as any person between ages 10 and 19 years. It’s a transitional stage of growth and development during which young people experience changes following puberty [1]. Since during this phase people become sexually active, healthy sexual awareness is mandatory for the future health status of the adolescents [2, 3]. Adolescents will develop different risky sexual behaviors, if they are unaware of healthy sexual behavior [3–5]. Some of risky sexual behaviors are having multiple sexual partners, sexual intercourse with commercial sex workers, unprotected sexual intercourse, coerced sexual intercourse and sexual intercourse for reward [6].

According to different studies, sexual risk behavior affects adolescents and youth life style and also contributes to different adverse effects, but its prevalence is rising due to many factors including lack of information regarding adolescent sexuality. Based on the UNAIDs report in 2013; globally there were 35 million people living with HIV, among which, youth between the ages of 15–24 years accounted for approximately 33% of new HIV infections. The WHO report showed that 333 million new cases of STIs occur globally each year, and at least 111 million of these cases occur under 25 years old peoples [6–8].

Studies revealed that sub Saharan countries including Ethiopia are the most affected. According to the family planning guidance association of Ethiopia, about 72% of boys and 71% of girls have had their first sexual contact within the age range of 15–17 yrs. while 13% of them started sexual activity between 10 and 14 yrs. of age. Analysis of 2002 in the country showed that 54% of pregnancy to girls under age 15 is unwanted as compared to 37% for those ages 20–24. According to these studies, major predisposing factors for raise of sexual activity among adolescents are increased pre-marital sex, deterioration of traditional norms & values that discourage premarital sex & media that transmit new ideas via movies. This indicates that the need to refocus programs and prioritize interventions tailored to adolescents under the age of 15 years [5, 9, 10].

As the study conducted in USA; the magnitude of risky sexual behavior was 46%, while in Ethiopia risky sexual behavior among secondary and preparatory school students was progressively increasing, for instance it was reported to be 15% in Jiga and 33% in Jimma [11–14]**.** A study conducted in Gonder city showed that, 20% of sexually active respondents ever had more than one sexual partner concurrently. Furthermore 16.7% of all sexually active male respondents had sexual contact with commercial sex workers, among those 81.2% of them never used condom. In Shendi town, north Ethiopia showed that, 19% of the study participants have had premarital sexual intercourse at the time of the survey, from which 7.6% had sexual experience with commercial sex workers. Another study conducted in Boditti, South Ethiopia showed that, about 21% of the respondents who sexual intercourse had it with two and more sexual partners in their lifetime [15–17].

Many investigations have been conducted on risk sexual behavior in different population groups, however it was neglected among young secondary school students in the study area. Despite different stakeholders’ effort to create awareness and to reduce STI, the rate of contracting STIs including HIV/AIDS is at an increasing rate [18]. Therefore, this research aimed to assess the school adolescent’s knowledge, attitude and practice of risky sexual behavior. The study will provide pertinent data on knowledge, attitude and practice of adolescents’ risky sexual behavior and it will give directions to tackle the problem of risky sexual behaviors in adolescents. Moreover, this research will provide better information for the governmental and NGO working on adolescents to improve their health both nationally and internationally. The study will also be a baseline by providing information for other researchers who want to conduct further surveys on this area and contribute to the development of the nation.

## Methods

### Study area and study period

The study was conducted in Metu secondary and preparatory school in Metu town, Oromia region, south western Ethiopia. Metu town is located 606KM away from Addis Ababa and 265Km from Jimma town. The town possesses three kebeles (which is the smallest administrative unit in Ethiopia); namely: 01, 02 and 03 and has an estimated total population of 46,810, from whom 23,786 are male and 23,024 are females. The main source of incomes of the indigenous community is agriculture. Metu is also known for honey and coffee production. The town has three secondary schools and two preparatory schools. This study was conducted among Metu secondary and preparatory school adolescents. The study was conducted from 04Feb-07June/2019.

### Study design

Institution based cross- sectional study was conducted.

### Source of population

All secondary and preparatory school adolescents in Metu town who were attending their class in 2019.

### Study population

All Metu secondary and preparatory school adolescents attending the class during the school hour.

### Inclusion criteria and exclusion criteria

#### Inclusion criteria

All students who have been present during the time of data collection at Metu Secondary and Preparatory School.

#### Exclusion criteria

Those who were seriously ill to the extent of unable to respond during the data collection period.

### Sample size determination

A single population proportion formula was used to estimate the sample size. The following assumptions were made while calculating the sample size. The degree of precision or margin of error chosen to be 0.05 with the reliability coefficient of 1.96% certainty (Z = 1.96). The prevalence rate of Risky Sexual Behaviors among Adolescents of Jimma University Community High School was 54.6% (21). The final sample size for this study with 10% non-response rate was a total of 362 students and systematic random sampling technique was used.

### Sampling procedure

**Fig. 1:**
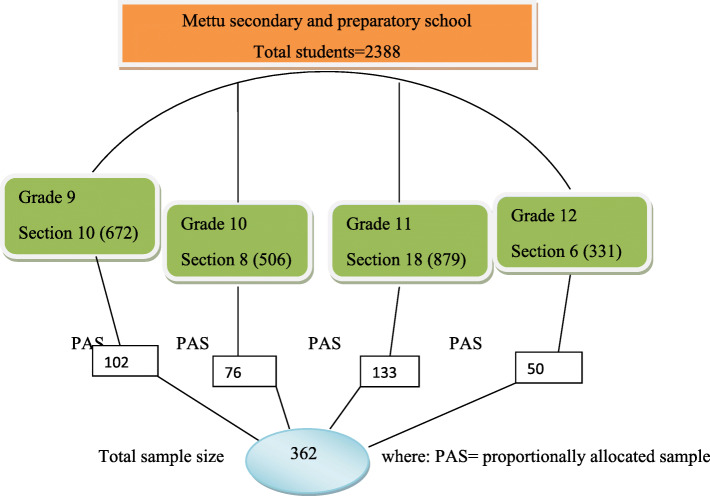
Schematic presentation of sampling procedure

A total of 2388 students are found in Metu secondary and preparatory school. A sampling frame which contained the lists of all students under each grade level (9th, 10th, 11th and 12th) was developed based on the lists obtained from students’ record office. Then the total sample size (362) is proportionally allocated for each grade levels as follows **(**Fig. 1) and after calculating ‘K’ value, a systematic random sampling method was used to select the participants.

### Data collection tools and method

Data was collected by using structured, pretested and self-administered questionnaires from March 15–16 / 2019, which is adapted and modified from different reviewed literature (15–16, 18). The question and statements were arranged according to particular objectives that they could address.

After revision, the questionnaires which were prepared in English were translated to local language (Afan Oromo) and again then backed to English to ensure the similarity of its message. Pretest was conducted with 5% of sample size at Abdii borii secondary and preparatory school students before the actual data collection was conducted. After checking of questionnaires completeness with a pretest, the questionnaires are handed out to the students who consented to participate in the study and after securing informed consent, the actual data collection was conducted by using self-administered structured questionnaires. Fourth year Nursing Students collected the data and were principal investigators responsible to lead the data collection process.

### Operational definitions and definitions of terms

#### Knowledge

Questions were delivered to study participants in “Yes” or “No” options, and each correct response was given a score of 1 and a wrong answer given a score of 0. The total knowledge score ranged from 0 to 16. We computed the mean score to determine the overall knowledge of sexual risk behaviors of respondents. Those who scored the mean score and above are considered as having a good knowledge, whereas those respondents who scored below the mean score were categorized as poor in knowledge of risky sexual behavior.

#### Attitude

We assessed the attitude using a 2 point Likert scale. In the scoring system that was used: Disagree =0 and Agree = 1. The total attitude score ranged from 0 to 8. Then we calculated the mean score. Those who scored the mean score and above are considered as having a positive attitude, whereas those who scored below the mean score were categorized as negative in attitudes towards risky sexual behavior.

##### Risky sexual practice

Those respondents who had sex before 18 years, used condom inconsistently and had multiple sexual partners.

### Data processing and analysis

Data was cleaned, coded and then entered into the computer using EpiData and exported into SPSS 21th version for analysis. The finding was presented by statements, graphs and tables.

## Results and discussion

### Results

#### Socio-demographic characteristics

A total of 361 study subjects participated in this study with a 99.7% response rate and among the total participants, 171(47.4%) were females and 1190 (52.6%) were males. The male to female ratio is 1.1:1. All of the participants responded to the questionnaires accordingly. Majority; 289 (80.01%) of the respondents were in the age range of 15–19 years (Table 1).

**Table 1:** Socio-demographic characteristics of Metu secondary and preparatory school adolescent students; Metu town, south western Ethiopia; June, 2019

SNo	Characteristics		Frequency	Percent
1	Sex	Male	190	52.6
		Female	171	47.4
2	Age	< 15	72	19.9
		15–19	289	80.1
3	Grade	9-10th	179	49.6
		11-12th	182	50.4
4	Place of primary school	Urban	242	67.0
		Rural	119	33.0

Among the participants 242 (67.0%) have attended their primary school in the urban areas while the rest 119 (33.0%) have attended their school in the rural areas. Of the total respondents; 290(80.3%) respondents were living with their parents (mother and father) and the rest 71 (19.7%) were living with their grandparents, father alone, mother alone, brothers or sisters.

**Fig. 2:**
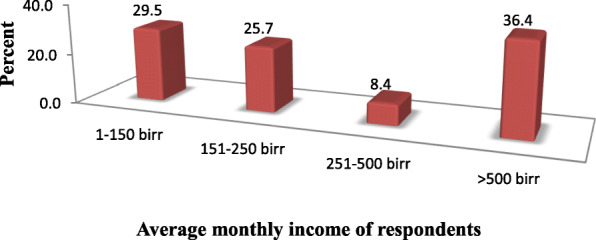
Average monthly income of adolescent students of Metu secondary and preparatory school; Metu town, south western Ethiopia; June, 2019

Regarding occupation of the respondents keepers; 157 (43.5%) were farmers; while 89 (24.7%), 75 (20.8%) and 26 (7.2%) were governmental organization employees, merchants and NGO employees respectively. Majority of the respondents keepers 339(93.9%) has an average monthly income which is greater than 750 birr, while the remaining 22(6.1%) has an average monthly income which is less than 750 birr. From a total of 361 respondents, 275 (76.2%) get pocket money from different sources. For instance 226 (82.2%) of them get pocket money from their parents, 19 (6.9%) from their boy or girl friends and the rest 30 (10.9%) of the respondents get pocket money from their relatives and siblings (Fig. 2).

#### Knowledge related to risky sexual behaviors

Among the total respondents, 274 (75.9%) of them has awareness about risky sexual behavior, from these, 139(50.7%) of them defined risky sexual behavior as unprotected sex; 136(49.6%) defined it as sexual practice after taking alcohol, drugs and chat; 129(47.1%) defined it as sexual practice with multiple sexual partners; whereas 113(41.2%), 101(36.9%) and 73(26.6%) of them defined it as having sex before marriage, sexual practice with incomparable partner and unusual sexual practice like anal and oral sex respectively. About 276 (76.5%) of the respondents have an awareness over the consequences of unsafe sex. Specifically, among the respondents who had an awareness over the consequence of unsafe sex; 242(87.7%) of them responded that unsafe sex has the consequence of STIs including HIV/AIDS which is followed by unwanted pregnancy 205(74.3%) (Table 2).

**Table 2:** Awareness on consequence of unsafe sex among Metu secondary and preparatory school adolescent students; Metu town, south western Ethiopia; June, 2019

Sr. No	Characteristics	Frequency	%
1	Unwanted pregnancy	205	74.3
2	Abortion	161	58.3
3	STIs including HIV/AIDS	242	87.7
4	Social stigma	154	55.8
5	Psychological problems	140	50.7
6	Educational interruption	150	54.3

With Regards to participants source of information about sexual matters; about 222 (61.5%) of the participants had heard about sexuality from friends; 164 (45.4) had heard about sexuality from teachers and 154 (42.7%) had heard about sexuality from parents, 101(28%) had heard about sexuality from magazine and 187(51.8%), 154(42.7%) had heard about sexuality from media and health personnel respectively. Most of the respondents 225(62.3%) have poor knowledge regarding risky sexual behavior and the remaining 136(37.7%) have good knowledge regarding risky sexual behavior.

#### Attitude related to risky sexual behaviors

About 254 (70.4%) of the respondents responded as they agree on substance use (like cigarette, alcohol, hashish) can expose to risky sexual behaviors and 252 (69.8%) believes that peer pressure enforces adolescents to undergo unprotected sex. While 166(46.0%) of the respondents agree that it is possible for girls to remain virgin until marriage (Table 3).

**Table 3:** Attitude towards risky sexual behavior among Metu secondary and preparatory school adolescent students; Metu town, south western Ethiopia; June, 2019

S/no	Statement	Agree	Disagree
1	It is possible for girls to remain virgin until marriage	166(46.0%)	195(54%)
2	Boys put girls under pressure to have sex	186(51.5%)	175(48.5%)
3	Condoms are not good for adolescents because it encourage them to have sex	153(42.4%)	208(57.6%)
4	Condoms reduce sexual pleasure	197(54.6%)	164(45.4%)
5	It is possible to talk with parents about sex	215(59.6%)	146(40.4%)
6	Girls don’t use condom because they trust their partners.	165(45.7%)	196(54.3%)
7	Substance abuse like khat, cigarette, alcohol, hashish can expose to risky sexual behavior	254(70.4%)	107(29.6%)
8	Peer pressure enforces adolescents to undergo un protected sex.	252(69.8%)	109(30.2%)

In contrast, for different reasons (challenges); 195 (54%) of the respondents disagreed with the statement that it is possible for girl to remain virgin until marriage. As to the challenges for this; 65 (40.6%) of the respondents mentioned that peer pressure is the major factor for this difficulty and 33 (20.6%) respondents mentioned substance abuse, 28 (7.8%) rape, 22(6.1%) respondents mentioned high sexual desire, 5 (3.1%) respondents mentioned weak academic potential and the rest respondents mentioned economic dependency & abduction as the challenges for a girl to remain virgin until marriage. Among the total respondents, 186(51.5%) respondents have positive attitudes towards risky sexual behavior while the remaining 175(48.5%) respondents have negative attitudes towards risky sexual behavior.

#### Practice concerning risky sexual behaviors

Among the total respondents; 194 (53.7%) of respondents have boy or girl friends while the rest 167 (46.3%) respondents have no friends for the moment. From the boy or girl friends of the respondents, 86(44.3%) of them are found under 18 years old, 105(54.1%) of them are found between the age range of 19–29 years old; while the rest 3(1.5%) of them are greater than 30 years old. The boy or girl friends of the respondents had different professions (Table 4).

**Table 4:** Participants of the study who had boy/girlfriend during the study period among Metu secondary and preparatory school adolescent students; Metu town, south western Ethiopia; June, 2019

S. No	Characteristics	Response	Frequency	
			No.	%
1	Do you have boy or girl friend?	Yes	194	53.7
		No	167	46.3
2	Occupation of your boy or girl friend	Students	125	64.4
		Driver	12	6.2
		Teacher	20	10.3
		Merchants	19	9.8
		Other	18	9.3

Of 81 (22.7%) respondents who had practiced sex; 49 (60.5%) were males and 32 (39.5%) were females. The rest 280 (77.3%) respondents were still virgin or had not practiced sex yet. Among the respondents who had practiced sex;14(17.3%) of them had practiced their first sexual intercourse at the age greater than or equal to 19 years, but most of them had their first sexual intercourse within the age range of 15–18 years which accounts for 51(63%). From this figure, males account for 38 (46.9%) and 27 (33.3%) for females. The remaining 11 (13.4%) males and 3 (3.7%) females had their sexual intercourse between the age of 10–14 years and only 2 (2.5%) female respondents had sexual intercourse before the age of 10.

Out of the respondents who had sexual intercourse; 57(70.4%) said their first time sex with students, 9 (11.1%) of them girl or boy friend’s, 3(0.8%) of the respondents had sex with their teachers and the rest 4 (15.8%) of the respondents had sex with their close friends and 8(2.2%) of the respondents had sex with married women or married men.

**Fig. 3:**
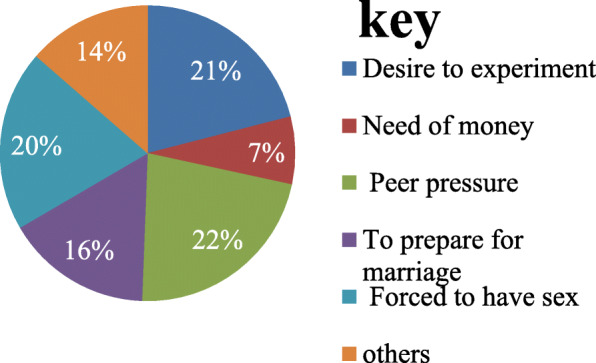
Participants reason for their first sexual exposure among Metu secondary and preparatory school adolescent students; Metu town, south western Ethiopia; June, 2019

**Fig. 4:**
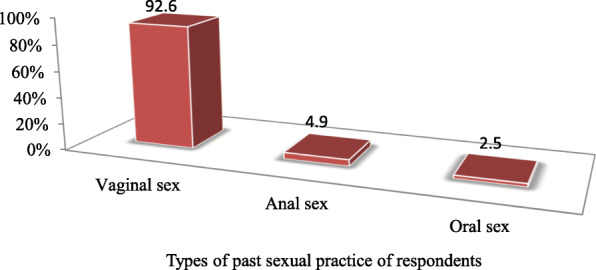
Types of past sexual practice among Metu secondary and preparatory school adolescent students; Metu town, south western Ethiopia; June, 2019

The major factors that initiated respondents for their first sexual intercourse were peer pressure which accounts 18(22.2%), 17(21.0%) respondents were initiated by the desire to practice sex; while 16(4.4%) of them are engaged in to sex by force **(**Fig. 3). Majority (92.6%) of the respondents were practiced vaginal intercourse/sex (Fig. 4).

From a total of 81 respondents who had ever sex; 50 (61.7%) of respondents had more than one sexual partners, of which male accounts for 54.2% and female accounts for 45.8% and the rest 31 (38.3%) respondents had only one sexual partner.

From those who had ever sex, 16 (19.8%) had always used condom during their sexual intercourse with their partners, but about 18 (22.2%) of them uses condom sometimes and 47(58%) of them had never used condom during sexual intercourse. From those who sometimes or never used condoms during their sexual practices, 14 (41.8%), 7 (20.6%) and the 13 (38.2%) of respondents were tested for HIV, pregnancy and other STIs respectively.

**Fig. 5:**
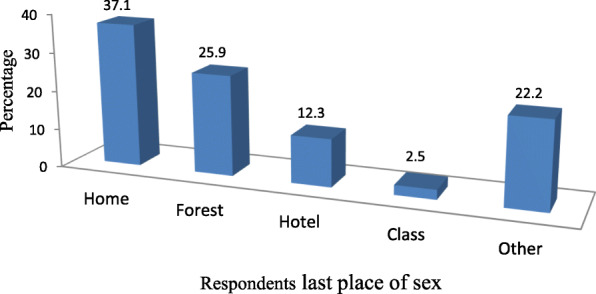
Place of respondents last sexual intercourse among Metu secondary and preparatory school adolescent students; Metu town, south western Ethiopia; June, 2019

Among those who tested for HIV; 12 (85.7%) of them were negative while 2 (14.3%) of them were positive and all of them are on ART. From females tested for pregnancy 6 (85.7%) were negative for pregnancy whereas 1 (14.3%) were positive and conducted abortion at health institution. From the respondents who were tested for STI, 10(76.9%) were found to be negative for STIs and the rest 3 (23.1%) were positive. Respondents who ever had practiced sex used different places for their last sexual intercourse. For instance, 30(37.04%) of them at home, 21 (25.9%) practiced in forest and 10 (12.3%) in at hotel (Fig. 5).

## Discussion

Many studies have been conducted on risk sexual behavior in different population groups in different parts of the world including Ethiopia, however the issue was neglected among young secondary school students in the study area. Therefore this study aimed to assess knowledge, attitude and practice towards risky sexual behavior among adolescents.

The study has found that 76.5% of respondents have awareness about the consequences of risky sexual behaviors. This finding is lower than a study conducted in Jimma; at which about 85% of the respondents knew consequences of risky sexual behavior [14] and higher than a study conducted in Nepal 67% [19]. This difference might be due to information source gaps, difference in educational background of the respondent’s parents and socio demographic difference.

The result of this study revealed that about 43% of respondent’s source of information about sexual matters were the parents. It is high as compared to a study done in Nigeria 11% [20] and Zambia 7% [21]. This dissimilarity might be due to lack of open communication between parents and adolescents about sexual matters.

According to the result of this study about 38% had good knowledge about risky sexual behaviors. This is lower than finding of a study in Jimma 77% [14], Bophuthatswana 70% [23]. This difference might be due to the difference on cut point, the nature of questions that respondents were asked, and study populations from different cultural and socio demographic backgrounds.

From the total study respondents, about 52% of them have a positive attitude towards risky sexual behavior which is in line with the study conducted at Jimma; in which 55% of them had a positive attitude towards risky sexual behavior [14]. This disparity might be due to not having communication about sexual matter with their parents, as result most information for their scattered knowledge comes from peers of the same sex who themselves lacks adequate information about reproductive health [23].

Based on the findings of this study, 23% of the respondents had ever practiced sexual intercourse which is lower in comparison with a study done in USA 46% [11, 12], Jimma 33% [14], Boditti, south Ethiopia 29% [17], higher than a study done in Nepal 9% [19] and was in line with a study done in Kenya, Nairobi 22% [24]. This difference might be due to difference in study participant Socio demographic, difference in traditional and cultural background.

According to the findings of this study, peer pressure (22%) was the major reason/ factor that influenced the respondents for their first sexual intercourse. This finding is lower than a study done at Boditi, South Ethiopia 40% [17] and Nepal 34% [19]. This disparity might be due to dissimilarity in interest and satisfaction of the study subjects, and level of peer influence on sexual matters.

In this study, from a total of 81 respondents who had ever sex, 62% of them had multiple sexual partners which is higher as compared to a study conducted in USA 41%, Jiga 29%, Gonder city 20%, Shendi town, west Gojam 25%, boditi, South Ethiopia 21% and Nigeria 7% [11–13, 15–17, 20]. This dissimilarity may be due to the difference in community norm about multiple sexual partners.

Among the study participants who had committed sex (81), about 42% of them used condom when they had their last sex. This finding is lower than a study conducted at Nairobi, Kenya (89%) [24], Gonder city 67.6% [15], USA 61% [11, 12], higher than Nigeria 27% [20] and Jiga 17% [13]. This difference might be due to lack of trust or faithfulness, decreased pleasure, lack of knowledge of condom benefits, less fear of contracting HIV/AIDS as it can now be controlled by medication, influence of tradition and scarcity of female condoms and the perception by women that it is complicated to insert.

Since the study was on a very sensitive and private issue; the possibility of underestimation cannot be ruled out and the study is limited to school so that the result cannot be generalized to the whole adolescents in the study area and due to the nature of the study design may not show cause and effect so that it should be repeated with different study design.

## Conclusions

Even though the majority of the students have an awareness regarding sexual risk behaviors; considerable number of students have practiced risky sexual behaviors that might predispose them for different sexual and reproductive health problems. Peer pressure was revealed as a major factor that influences the respondents towards their first sexual intercourse. Peers have greater influence on the positive and negative behavior of their friends. Therefore the school should emphasize on promoting peer educators and peer discussion to protect adolescents and youth from risky sexual behaviors.

## Data Availability

The data that support the findings of this study is available at the hands of Mr. TK, the corresponding author and it can be delivered to the journal based on request at any time.

## Abbreviations

AAU: Addis Ababa University
ART: Antiretroviral therapy
CDC: Center of disease control and prevention
HIV/AIDS: Human immune deficiency virus/ Acquired immunodeficiency Syndrome
JUCMHS: Jimma University College of Medicine and health science
MOH: Ministry of health
NGO: Nongovernmental organization
STDs: Sexually transmitted diseases
STls: Sexually transmitted infections
WHO: World health organization
